# Serological Detection of *Toxoplasma gondii* among Free-Grazing Ducks from Central and Western Thailand—A One Health Perspective on Integrated Farming

**DOI:** 10.3390/tropicalmed8020103

**Published:** 2023-02-06

**Authors:** Thi Thuy Nguyen, Ketsarin Kamyingkird, Rungrot Jam-on, Waraphon Phimpraphai, Pun Panomwan, Adrian B. Hehl, Tawin Inpankaew

**Affiliations:** 1 Department of Parasitology, Faculty of Veterinary Medicine, Kasetsart University, Bangkok 10900, Thailand; 2 Department of Veterinary Medicine, Faculty of Animal Science and Veterinary Medicine, College of Agriculture and Forestry, Hue University, Hue 49000, Vietnam; 3 Department of Farm Resources and Production Medicine, Faculty of Veterinary Medicine, Kasetsart University, Nakhon Pathom 73000, Thailand; 4 Department of Veterinary Public Health, Faculty of Veterinary Medicine, Kasetsart University, Bangkok 10900, Thailand; 5 Faculty of Veterinary Medicine, Mahanakorn University of Technology, Bangkok 10530, Thailand; 6 Laboratory of Molecular Parasitology, Institute of Parasitology, University of Zurich, 8000 Zurich, Switzerland

**Keywords:** *T. gondii*, free-grazing ducks, IFAT, slaughterhouse, Thailand

## Abstract

Toxoplasmosis is one of the most common zoonotic parasitic diseases infecting nearly all warm-blooded animals, including poultry (geese, turkeys, chickens, and ducks). It is caused by *Toxoplasma gondii* (*T. gondii*), which is an obligate intracellular protozoan belonging to the Apicomplexa group. In Thailand, duck meat production for domestic consumption and international trade is mainly bred and produced in the central and western parts of the country. Free-grazing ducks in integrated duck–rice production have significant benefits in rice cultivation, accounting for the popularity of this farming system in Thailand. However, ducks are considered particularly susceptible to consuming *T. gondii* oocysts from water contaminated with cat feces due to the fact of their feeding habits of free-grazing and dabbling. Hence, the prevalence of this zoonotic parasite in a large-scale integrated farming context is particularly challenging with respect to the contamination of the food chain of humans and farm animals. In the present study, we examined the overall prevalence of *T. gondii* infection in slaughtered free-grazing ducks originating from Central and Western Thailand, setting the stage for an in-depth One Health approach to assess and manage the risks of integrated farming practices. A representative sample size of 161 ducks was calculated using a two-stage sampling method. Specifically, serum samples were collected from 217 slaughtered free-grazing ducks originating in six provinces in Central and Western Thailand. Serum antibodies against *T. gondii* were detected using an indirect fluorescent antibody test (IFAT). The positive control serum samples were prepared from ducks experimentally immunized with *T. gondii*. Sixty-eight (31.3%) of the two hundred and seventeen ducks were seropositive with *T. gondii*. Two groups of fattening ducks and spent layers showed similar seropositivity rates at 29% and 32.3%, with the majority of positive samples being found in the low titer. In addition, a wide distribution of positive serum samples was observed in all six provinces in the present study. To the best of our knowledge, this is the first report on a serological prevalence snapshot in commercially produced duck populations that have high interaction with farmed environments in Thailand, revealing a high infection pressure in areas of integrated duck–rice farming. Importantly, contaminated duck meat for commercial use, as well as offal and carcasses from slaughterhouses, completes the transmission of *T. gondii* from the environment into the food chain of humans and domestic animals. Hence, from a One Health perspective, it is important to clarify whether this transmission chain extends further to the wild, i.e., predator–prey cycles that are independent of duck farming or are self-contained.

## 1. Introduction

*Toxoplasma gondii* (*T. gondii*) is an obligate intracellular protozoan causing toxoplasmosis which is of significant medical and veterinary importance. Felids are the only definitive hosts producing environmentally resistant, highly infectious oocysts, which are shed with feces and survive for up to two years in the environment. Conversely, intermediate hosts of *T. gondii*, consisting of all warm-blooded animals, are of great significance in harboring infectious tissue cysts [1].

A systematic review previously revealed an average global *T. gondii* seropositivity rate of 25.7% in humans over the last three decades [2]. Water and food contaminated with *T. gondii* oocysts or undercooked meat of chronically infected animals containing tissue cysts can become sources of infection for humans [1]. Healthy individuals infected with *Toxoplasma* are typically asymptomatic during the self-limiting acute phase and become chronically infected for life, whereas some such clinical signs as cervical or occipital lymphadenopathy, microphthalmia (ocular toxoplasmosis), encephalitis, and hydrocephaly or microcephaly (congenital toxoplasmosis) can be seen in immune-compromised patients [3,4].

In Thailand, *T. gondii* infections have been reported in various animal species but lack a study in ducks [5,6,7,8,9,10]. Approximately 2.27 M metric tons of Thai duck meat products, including cooked, refrigerated, frozen, and otherwise treated duck meat, were exported to Japan, Hongkong, Europe, South Africa, Bangladesh, and neighboring countries, such as Myanmar, Laos, and Cambodia, in 2021 [11,12]. Central and Western Thailand are the major areas for producing duck meat and eggs for domestic consumption and international exportation [13,14].

Thailand is the world’s second-largest rice exporter, with production mostly in the central plains aided by local irrigation systems and yielding two or even three crops each year. Rice production is traditionally linked to “ped lai thoong”, the co-cultivation of Khaki Campbell ducks released in large flocks to feed in freshly harvested paddies. This development of rice cultivation has been closely associated with the free-grazing duck farming system in which large flocks of ducks are rotated among paddy fields after the harvest to scavenge leftover rice grains, husks, insects, small fishes, and snails for ducks [14]. This is beneficial for rice production in several ways including lowering the requirement for combating pests. Fattening ducks, reared for meat production only, are usually slaughtered after two months in the paddies. In addition, ducks are raised for egg production. They are kept in paddy fields for 5–6 months prior to a nesting period of one to two years at farms and slaughtered as spent layer ducks for meat [14,15]. Hence, because of their feeding habits free-grazing ducks are highly exposed to *T. gondii* oocysts while grazing in large areas in these irrigated environments. The prevalence of seropositivity in these ducks can thus be considered a reliable indicator of the infection pressure by *T. gondii* in the farmed environment. As a consequence, the consumption of undercooked duck meat constitutes a risk for *T. gondii* infection in humans and animals [1,16,17]. From a One Health perspective, environmental and animal health are the relevant focus areas of this system at first glance. However, the fact that the waterborne zoonotic pathogen *T. gondii* enters the human food chain via duck meat, contaminated with tissue cysts containing infectious bradyzoites, adds human health as the third pillar of the One Health concept. The consequences for human health as a result of this particular form of integrated farming remains to be determined. Ultimately, we want to bring to bear a systems approach to assess and evaluate this problem and use this approach to design strategies for intervention. As a first assessment of the importance of this medical and economical threat, we conducted a prevalence snapshot in commercially reared free-grazing duck populations at the time of slaughter to determine the presence of antibodies against *T. gondii* in ducks from areas of Central and Western Thailand.

## 2. Materials and Methods

### 2.1. Study Design and Sample Size Calculation

Khaki Campbell ducks raised in the central and western parts of Thailand were included in this study. The fattening ducks and spent layers, which are typically raised in the traditional free-grazing system, were collected. To detect antibodies to *T. gondii* in the free-grazing ducks, a sample size calculation was performed using a two-stage sampling method: $n_{h/i}$ = $\left[1-{\left(1-\mathrm{CL}\right)}^{1/e}\;\right]\times \left(N_{h/i}-\frac{e-1}{2}\right)$, where CL is the level of confidence, at 95%; e is the number of detectable individuals with the event in the population: e = N × p × Se, with p being the expected prevalence of 15% at the individual level [18] and 32% at farm level [19], and Se being the sensitivity of IFAT, at 80% [20]; Nh/i is the number of flocks/individuals per flock within 2 months (estimated sampling period in a slaughterhouse), at 90 flocks/1000 ducks; and nh/i is the number of flocks/individuals per flock. A minimal sample size calculation was seven flocks and 23 ducks/flock. Before stunning, blood samples from individual birds were collected in a local slaughterhouse located in Nakhon Pathom province.

### 2.2. Blood Sample Collection

Between April and May 2021, the blood was sampled in the duck superficial plantar metatarsal vein using serum tubes (BD, Belliver Industrial Estate, Plymouth, UK) by sterile 3–5 milliliter syringes with 20–21# needles. The sample tubes were left standing for at least 30 min at ambient temperature for coagulation before transporting them to the laboratory. The blood was then centrifuged at 1100× *g* for 5 min, and serum fractions were removed and stored at −20 °C for serological examination.

### 2.3. Positive and Negative Control Sera

*T. gondii*-positive and -negative duck sera were generated. Briefly, 500 microliters of 10^4^ tachyzoites of in vitro cultivated *T. gondii* PLK strain were intravenously inoculated into five Khaki Campbell ducks aged one month for the positive control. Negative control serum samples were collected from three ducks injected with 500 microliters of sterile PBS (phosphate-buffered saline, pH 7.4). All ducks were examined as clinically healthy and seronegative with *T. gondii* before inoculation. The serum samples were collected on the 1st, 2nd, 3rd, 4th, 5th, 6th, 7th, and 8th week postinoculation (wpi.). The positive and negative control sera were confirmed by an inhouse indirect fluorescent antibody test (IFAT) (see Section 2.4 below).

### 2.4. Serological Examination

An inhouse qualitative indirect fluorescent antibody test (IFAT) was used for the detection of *T. gondii* antibodies in the duck serum samples. Preparation of IFAT slides: *T. gondii* tachyzoites of the RH strain were cultivated in vitro in African green monkey kidney (Vero) cells [21,22]. The monolayer of the infected cells was harvested, and *T. gondii* tachyzoites were released and enriched using several passages through syringe needles and a 5 μm filter. The tachyzoites were then washed three times with cold PBS and diluted to 10^6^ tachyzoites/mL. Twenty microliters of a *T. gondii* tachyzoite suspension were pipetted into each well of Teflon-coated slides (Cel-Line Associates, Newfield, NJ, USA). The slides were then air-dried at room temperature overnight and fixed with ice-cold acetone for 10 min and air-drying before storing at −80 °C until used.

Detection of anti-*T. gondii* antibodies in duck serum: The serum samples were titrated at a two-fold dilution, starting at 1:25, in diluting buffer (NaH_2_PO_4_, 0.11 g; bovine serum albumin, 5 g; Na_2_HPO_4_, 0.595 g; NaCl, 4.275 g; and ddH_2_O, 500 mL) before and incubated with acetone-fixed tachyzoite-coated slides. The secondary antibodies (fluorescein-labeled antibody to duck IgG (H + L) generated in goats (KPL, Gaithersburg, MD 20878, USA)) were used at a dilution of 1:150. The positive and negative control sera were included in each test. The slides were incubated with diluted serum or secondary antibody solutions at 37 °C for 30 min and washed three times with FA Rinse buffer (Na_2_CO_3_, 1.14 g; NaHCO_3_, 3.36 g; NaCl, 0.85 g; and ddH_2_O, 1000 mL) before embedding under coverslips. The slides were examined microscopically under fluorescence light. Only a bright, linear peripheral fluorescence of the tachyzoites was considered positive. A titer of 1:100 was used as the positive cut-off value for *T. gondii* [19].

### 2.5. Statistical Analyses

The statistically significant difference between the frequencies of the seropositive samples in the two duck types was considered at *p* < 0.05 using the χ^2^ test. The normal distribution of antibody titers was determined using the Shapiro test, and the Wilcoxon’s test was then employed to determine the statistically significant difference in the antibody titer distribution between the two duck types. These statistical analyses were conducted using the R software package version 3.6.3.

## 3. Results

### 3.1. Samples, Geographical Areas, and Data Characteristics

**Figure 1:**
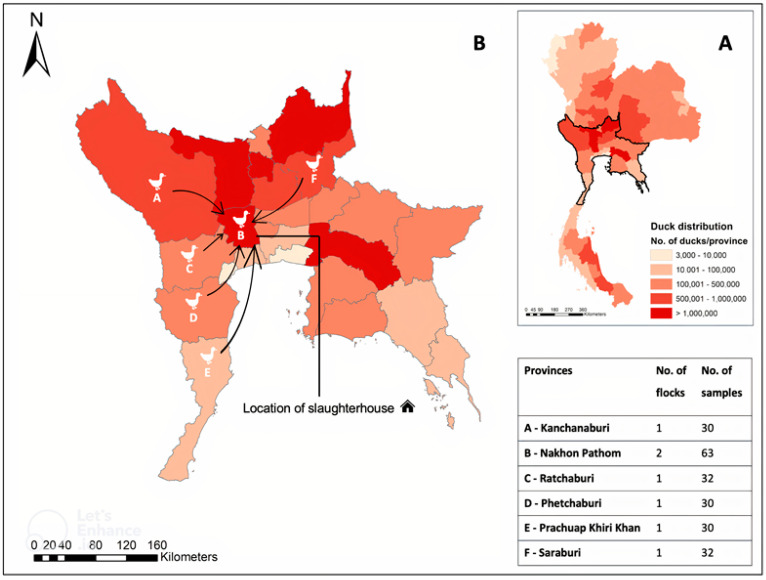
Study area: (**A**) distribution of free-grazing ducks in Thailand (13); (**B**) six investigated provinces with the number of flocks and ducks collected.

The antemortem serum samples were conveniently collected from 217 Khaki Campbell ducks (at least 30 ducks/flock) at the slaughterhouse. Five flocks of spent layers and two flocks of fattening ducks originated from six different provinces, including two flocks in Nakhon Pathom and one flock in each other provinces (Kanchanaburi, Phetchaburi, Prachuap Khiri Khan, Ratchaburi, and Saraburi) (Figure 1).

### 3.2. Seropositive Rate and Geographical Distribution of T. gondii Infection in Free-Grazing Ducks

Positive *T. gondii* antibody titers were detected in 68/217 (31.3%) ducks, with a titer of 1:100 being used as the cut-off value. The seropositive rates of *T. gondii* in the fattening and the spent layers were 29% and 32.3%, respectively. No significant variation was found between the two duck groups (𝜒^2^ = 0.1, df = 1, *p* = 0.8). The majority of the positive samples showed a low IFAT titer of 1:100 (75%), followed by a titer of 1:200 (23.5%). The Wilcoxon’s test showed a significantly higher titer distribution in the fattening ducks compared to the spent layers (W = 326.5, *p* = 0.02). Eighty-two % of the positive sera were observed at the titer of 1:100 in the spent layers, making it the most frequent titer in this type of duck. The titer of 1:200 accounted for the remaining 18% of the positive sera. Meanwhile, over 55% of the positive sera of the fattening ducks were recorded at a 1:100 titer. Only one sample had a titer of 1:400, and 38.9% of positive samples showed a 1:200 titer in this group (Table 1).

**Figure 2:**
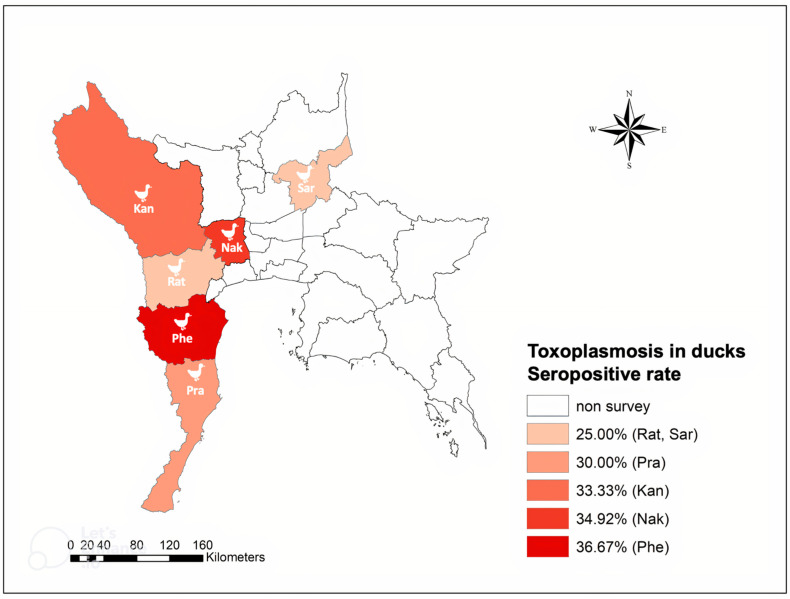
Geographical distribution of *T. gondii* infection in free-grazing ducks in the central and western areas of Thailand.

The seropositive ducks were widely distributed among all investigated provinces: Kanchanaburi, 10/30 (33.3%); Nakhon Pathom, 22/63 (34.9%); Phetchaburi, 11/30 (36.7%); Prachuap Khiri Khan, 9/30 (30%); and Ratchaburi and Saraburi, 8/32 (25%) (Figure 2). The differences in the seropositive rates of the flocks from the investigated provinces were not significant (*p* = 0.8).

## 4. Discussion

The present investigation revealed a high seropositivity rate for *T. gondii* (31.3%) in free-grazing ducks from the central and western provinces of Thailand compared to that in ducks from different sites throughout the world; varying seropositivity rates of *T. gondii* infection in ducks were also reported at 5.7% in Germany [19], 14.6% in Malaysia [18], 6.6–13.2% in China [23], and 10.6–55% in Egypt [24,25,26]. These differences might be due to the presence of technical or sampling differences (serological tests, cut-off values, sources for animals, sample sizes, age, feeding range, etc.) but also the magnitude of infection pressure from *T. gondii* oocysts or tissue cysts in animal carcasses (e.g., rodents) in the study areas (Table 2). The cut-off value of 1:100, which is considerably higher than those of previous studies, was chosen to improve the specificity of IFAT in the present study, thus avoiding false positives during the examination [19].

In Thailand, the tradition of rotating free-grazing ducks is closely linked to rice crop cycles and is widely distributed and commercialized throughout the central, western, and eastern regions [14]. Free-grazing duck flocks forage in the paddy fields until the flowering stage and then again after harvest time. They scavenge for insects, snails, husks, weeds, and leftover grains, thus reducing the need for chemical fertilizers and pesticides. Since no biosecurity aspects are considered in the free-grazing systems, grazing and dabbling ducks are constantly exposed to peroral transmission of waterborne pathogens from the environment [15]. In the case of *T. gondii*, this risk for individuals in a flock is cumulative with several dimensions, e.g., total surface area used for feeding (including transports of flocks to remote paddies), cumulative time of exposure, degree of oocyst contamination in feeding areas (linked to the density of the cat population), and management of offal from local slaughtering (use as cat food). This could be a reason why the free-grazing ducks in this study were relatively highly seropositive with *T. gondii* than caged or farm ducks. Indeed, they can ingest *T. gondii* tissue cysts in carcasses of intermediate hosts or oocysts released by cats in the fields, which in turn might indicate environmental contamination [19]. Moreover, the flocks are transported to other villages, districts, or even provinces over substantial distances after the exhaustion of the food supply in the paddy fields, perhaps also contributing to a wider distribution of oocysts [15]. In the present study, a similar significant prevalence of *T. gondii* infection in the free-grazing ducks sampled in all investigated provinces of Central and Western Thailand suggested the strong possibility of exposure to the parasite of the livestock and humans in these regions. Hence, to address these risks, a One Health concept is required, which takes all aspects of this integrated farming system into consideration [31]. This includes also abiotic environmental factors, such as rainfall and temperature. These climatic factors govern the circulation *T. gondii* oocysts in the natural environment [32,33]. A tropical savanna climate is recorded in the central and western parts of Thailand, with the annual rainfall and temperature being approximately 1390.8 mm and 29 °C, respectively. Especially, the highest mean temperature of approximately 36 °C, monthly precipitation of 188 mm, and relatively high humidity of 73% were recorded during the sample collection between April and May [34]. This humid and hot condition suits *T. gondii* oocysts to sporulate and prolong [32], thus increasing the risk of infection with sporulated oocysts in animals when feeding in the pastures.

Even though there was no statistically significant difference between the frequencies of positive sera in the two duck types, significantly higher antibody titers against *T. gondii* infection were found in the fattening ducks compared to the spent layer ducks in this study (*p* = 0.02). Maksimov et al. (2011) experimentally inoculated ducks with tachyzoites and oocysts of *T. gondii*, which revealed that their antibody response levels were partly dependent on the stages of the parasite with which the ducks were infected [19]. Indeed, the group inoculated with *T. gondii* oocysts developed stronger and longer-lasting antibody responses than those injected with tachyzoites. Additionally, antibodies to *T. gondii* were firstly observed in the first week postinoculation (wpi), and they reached the highest levels between the third and fourth wpi in both groups of duck. The antibody responses could then last for over eleven wpi and decrease more or less rapidly, eventually persisting for life at the residual level [19,35], indicating that antibody levels also depend on when the ducks are diagnosed during the infectious period. In this study, the fattening ducks (males) were normally released in the paddy fields for approximately a couple of months until slaughtered. Meanwhile, the spent layer ducks were also rotated in the fields for around a half year. Then, they were slaughtered after a nesting period of one to two years at the farms [15]. In other words, the fattening was sampled much earlier (2 months) than the spent layers (over a year of nesting period) after their exposure to pathogens. Therefore, a general tendency for higher antibody titers could be seen in the fattening ducks.

The domestic ducks were demonstrated to be relatively susceptible to *T. gondii* infection [19,36]. Although the disease does not frequently develop symptoms in poultry, especially in the cases of avirulent *T. gondii* strains, tissue cysts may be still distributed in some parts of the duck body [36]. From the aspect of food safety, skeletal muscles and hearts are considered the most intensively infected tissues [1]. Furthermore, *T. gondii* cysts from tissues on day 28 postinfection were successfully isolated [36]. In the present study, the antibodies against *T. gondii* infection were detected in both the fattening and spent layer ducks at 3 months of age and over, indicating that cysts are likely to present in their tissues. Therefore, determining the presence of tissue cysts in free-grazing ducks and oocysts in the environment is necessary to clarify the possibility of zoonosis in Thailand. Indeed, integrative research requiring a combination of spatial, geographic, and other mathematical models, field and laboratory methods, and veterinary and medical practices is obviously needed to assess the risk factors. This is also crucial in devising preventive measures for toxoplasmosis transmitted from livestock and the natural environment to humans, particularly in sheltering and outdoor cat management policies.

## 5. Conclusions

In summary, the present study indicated a high seropositivity rate of toxoplasmosis, and it is widely distributed in free-grazing ducks from Central and Western Thailand. This study revealed a considerable risk of infection for livestock and humans via the environment contaminated with *T. gondii* in the study areas. Therefore, the potential risk of human infection should be avoided by the restriction of consuming undercooked duck meat. Further studies on the genetic characterization are needed to clarify which *T. gondii* strains are present in ducks reared in farmed environments in Thailand. Furthermore, a prospective longitudinal study in a free-grazing duck flock might indicate the impact of seasonal changes on *T. gondii* infection.

## Figures and Tables

**Table 1:** *T. gondii* seropositive free-gazing ducks in this study.

Type of Ducks	No. of Tested	No. of Positive (%)	Statistical Analysis	Titer (%)		
				1:100	1:200	1:400
Fattening	62	18 (29)	𝜒^2^ = 0.1, df = 1, *p* = 0.8	10 (55.6)	7 (38.9)	1 (5.6)
Spent layer	155	50 (32.3)		41 (82)	9 (18)	0 (0)
Total	217	68 (31.3)	-	75	23.5	1.5

**Table 2:** Worldwide seropositive rates of *T. gondii* in ducks.

Country	Source	Seropositive Rate	Test	Cut-Off	Reference
Thailand	S ^1^/FR ^2^	31.3	IFAT	1:100	This study
Malaysia	F ^3^/FR	14.6	MAT ^6^	1:6	[18]
China	FR	13.2	MAT	1:25	[23]
	C ^4^	6.6			
Iraq	M ^5^	56	LAT ^7^	1:2	[27]
Iran	M/FR	46	MAT	1:20	[28]
Germany	F/FR	5.7	ELISA^8^ _(SAG1)_^9^	0.14	[19]
Czech Republic	F	14	IFAT	1:40	[29]
Poland	F	21.2	MAT	1:40	[30]
Egypt	F	55	IHA ^10^	1:80	[24]
	S	13.6	MAT	1:25	[25]
	M	10.6	ELISA _(SAG2t)_^11^	0.014	[26]

^1^ Slaughterhouse; ^2^ free range; ^3^ farm; ^4^ caged; ^5^ market; ^6^ modified agglutination test; ^7^ latex agglutination test; ^8^ enzyme-linked immunosorbent assay; ^9^ protein used in test; ^10^ indirect hemagglutination; ^11^ protein used in test.

## Data Availability

Not applicable.

## References

[1] Webster J.P. (2010). Review of “Toxoplasmosis of Animals and Humans (Second Edition)” by J.P. Dubey. Parasite Vectors.

[2] Molan A., Nosaka K., Wang W., Hunter M. (2019). Global status of *Toxoplasma gondii* infection: Systematic review and prevalence snapshots. Trop. Biomed..

[3] Pleyer U., Gross U., Schluter D., Wilking H., Seeber F. (2019). Toxoplasmosis in Germany. Dtsch Arztebl Int..

[4] Wanachiwanawin D., Sutthent R., Chokephaibulkit K., Mahakittikun V., Ongrotchanakun J., Monkong N. (2001). *Toxoplasma gondii* antibodies in HIV and non-HIV infected Thai pregnant women. Asian Pac. J. Allergy Immunol..

[5] Chaichan P. (2017). Epidemiology of *Toxoplasma gondii* in Thailand. Ph.D. Thesis.

[6] Inpankaew T., Pinyopanuwut N., Chimnoi W., Kengradomkit C., Sununta C., Zhang G., Nishikawa Y., Igarashi I., Xuan X., Jittapalapong S. (2010). Serodiagnosis of *Toxoplasma gondii* infection in dairy cows in Thailand. Transbound. Emerg. Dis..

[7] Jittapalapong S., Inpankaew T., Pinyopanuwat N., Chimnoi W., Kengradomkij C., Wongnarkpet S., Maruayama S., Lekkla A., Sukthana Y. (2010). Epidemiology of *Toxoplasma gondii* infection of stray cats in Bangkok, Thailand. Southeast Asian J. Trop. Med. Public Health.

[8] Jittapalapong S., Sittisan P., Sakpuaram T., Kabeya H., Maruyama S., Inpankaew T. (2009). Coinfection of *Leptospira* spp and *Toxoplasma gondii* among stray dogs in Bangkok, Thailand. Southeast Asian J. Trop. Med. Public Health.

[9] Kengradomkij C., Kamyingkird K., Pinyopanuwat N., Chimnoi W., Jittapalapong S., Inpankaew T. (2018). Seroprevalence of *Toxoplasma gondii* from stray cats residing in temples, Bangkok, Thailand. J. Trop. Med. Parasitol..

[10] Tuntasuvan D., Mohkaew K., Dubey J.P. (2001). Seroprevalence of *Toxoplasma gondii* in elephants (*Elephus maximus indicus*) in Thailand. J. Parasitol..

[11] Tridge Top Export Destinations of Duck Meat from Thailand 2022. https://www.tridge.com/intelligences/duck-meat/TH.

[12] Volza Grow Global Duck Meat Exports from Thailand 2021. https://www.volza.com/p/duck-meat/export/export-from-thailand/.

[13] Department of Livestock Development (2017). Data from: Number of livestock inventory in Thailand. https://ict.dld.go.th/webnew/index.php/th/service-ict/report/289-report-thailand-livestock/reportservey2560.

[14] Gilbert M., Chaitaweesub P., Parakamawongsa T., Premashthira S., Tiensin T., Kalpravidh W., Wagner H., Slingenbergh J. (2006). Free-grazing ducks and highly pathogenic avian influenza, Thailand. Emerg. Infect. Dis..

[15] Songserm T., Jam-on R., Sae-Heng N., Meemak N., Hulse-Post D., Sturm-Ramirez K., Robert G.W. (2006). Domestic Ducks and H5N1 Influenza Epidemic, Thailand. Emerg. Infect. Dis..

[16] El-Massry A., Mahdy O.A., El-Ghaysh A., Dubey J.P. (2000). Prevalence of *Toxoplasma gondii* antibodies in sera of turkeys, chickens, and ducks from Egypt. J. Parasitol..

[17] Dubey J.P. (2010). *Toxoplasma gondii* infections in chickens (*Gallus domesticus*): Prevalence, clinical disease, diagnosis and public health significance. Zoonoses Public Health.

[18] Puvanesuaran V., Noordin R., Balakrishnan V. (2013). Isolation and genotyping of *Toxoplasma gondii* from free-range ducks in Malaysia. Avian Dis..

[19] Maksimov P., Buschtons S., Herrmann D.C., Conraths F.J., Gorlich K., Tenter A.M., Dubey J.P., Nagel-Kohl U., Thoms B., Botcher L. (2011). Serological survey and risk factors for *Toxoplasma gondii* in domestic ducks and geese in Lower Saxony, Germany. Vet. Parasitol..

[20] Casartelli-Alves L., Boechat V.C., Macedo-Couto R., Ferreira L.C., Nicolau J.L., Neves L.B., Millar P.R., Vicente R.T., Oliveira R.V.C., Muniz A.G. (2014). Sensitivity and specificity of serological tests, histopathology and immunohistochemistry for detection of *Toxoplasma gondii* infection in domestic chickens. Vet. Parasitol..

[21] Nguyen T.T., Kengradomkij C., Inpankaew T. (2020). Detection of antibodies to *Toxoplasma gondii* among owned dogs in Cambodia. Food Waterborne Parasitol..

[22] Sabin A.B. (1941). Toxoplasmic encephalitis in children. JAMA.

[23] Zhao G., Song Z., Wang S., Hassan I.A., Wang W., Cheng F., Yang X. (2015). A seroepidemiological survey of *Toxoplasma gondii* infection in free-range and caged ducks in Southwest China. Isr. J. Vet. Med..

[24] Harfoush M., Tahoon Ael N. (2010). Seroprevalence of *Toxoplasma gondii* antibodies in domestic ducks, free-range chickens, turkeys and rabbits in Kafr El-Sheikh Governorate Egypt. J. Egypt Soc. Parasitol..

[25] Aboulaila M., ElBahy N., Hilali M., Yokoyama N., Igarashi I. (2011). Serodiagnosis of *Toxoplasma gondii* in ducks from Behera Governorate, Egypt. J. Protozool. Res..

[26] Ibrahim H.M., Osman G.Y., Mohamed A.H., Al-Selwi A.G.M., Nishikawa Y., Abdel-Ghaffar F. (2018). *Toxoplasma gondii*: Prevalence of natural infection in pigeons and ducks from middle and upper Egypt using serological, histopathological, and immunohistochemical diagnostic methods. Vet. Parasitol. Reg. Stud. Rep..

[27] Alkhaled M.J.A., Yakoob A.Y., Al-Hamadani A.H.U. (2012). An investigation of Toxoplasmosis in Free Range chickens, Industrial chickens and Duck in mid Euphrates area of Iraq. AL-Qadisiya J. VetMedSci.

[28] Amouei A., Sharif M., Hosseini S.A., Sarvia S., Mizani A., Salehi S., Gholami S., Jafar-Ramaji T., Daryani A. (2018). Prevalence of *Toxoplasma gondii* infection in domestic and migrating birds from Mazandaran province, Northern Iran. Avian Biol. Res..

[29] Bártová E., Sedlák K., Literák I. (2009). Serologic survey for toxoplasmosis in domestic birds from the Czech Republic. Avian Pathol..

[30] Sroka J., Wojcik-Fatla A., Szymanska J., Dutkiewicz J., Zajac V., Zwolinski J. (2010). The occurrence of *Toxoplasma gondii* infection in people and animals from rural environment of Lublin region-estimate of potential role of water as a source of infection. Ann. Agric. Environ. Med..

[31] Adisasmito W.B., Almuhairi S., Behravesh C.B., Bilivogui P., Bukachi S.A., Casas N., Becerra N.C., Charron D.F., Chaudhary A., One Health High-Level Expert Panel (OHHLEP) (2022). One Health: A new definition for a sustainable and healthy future. PLOS Pathog..

[32] Yan C., Liang L.J., Zheng K.Y., Zhu X.Q. (2016). Impact of environmental factors on the emergence, transmission and distribution of *Toxoplasma gondii*. Parasit Vectors.

[33] Simon A., Poulin M.B., Rousseau A.N., Ogden N.H. (2013). Fate and transport of *Toxoplasma gondii* oocysts in seasonally snow-covered watersheds: A conceptual framework from a melting snowpack to the Canadian arctic coasts. Int. J. Environ. Res. Public Health.

[34] Thai Meteorological Department Automatic Weather System Climate Data. http://www.aws-observation.tmd.go.th/web/climate/climate_past.asp.

[35] Robert-Gangneux F., Dardé M.L. (2012). Epidemiology of and diagnostic strategies for toxoplasmosis. Clin. Microbiol. Rev..

[36] Bártová E., Dvoráková H., Bárta J., Sedlák K., Literák I. (2004). Susceptibility of the domestic duck (*Anas platyrhynchos*) to experimental infection with *Toxoplasma gondii* oocysts. Avian Pathol..

